# Eculizumab in severe pediatric STEC-HUS and its impact on neurological prognosis—a systematic review and meta-analysis

**DOI:** 10.1007/s00431-025-06160-2

**Published:** 2025-05-08

**Authors:** Rachele Spagnol, Alessandra Alfisi, Marco Moi, Ilaria Bonvecchio, Nicola Bertazza Partigiani, Enrico Vidal

**Affiliations:** 1 Pediatric Nephrology Unit, Department of Women’s and Children’s Health, University of Padua, Padua, Italy; 2 Department of Medicine (DMED), University of Udine, Udine, Italy

**Keywords:** Eculizumab, STEC-HUS, Neurological involvement, Neurological outcome

## Abstract

Hemolytic-uremic syndrome (HUS) is defined by the triad of microangiopathic hemolytic anemia, thrombocytopenia, and acute kidney injury (AKI) and is caused, in 90% of pediatric cases, by Shiga toxin-producing *Escherichia coli* (STEC-HUS) infection. While targeting complement component C5 using eculizumab has shown benefit in atypical HUS, its effect on STEC-HUS, especially on neurological outcome, remains unclear. This systematic review and meta-analysis aimed to evaluate the impact of eculizumab on neurological prognosis in pediatric STEC-HUS. The review was conducted in accordance with PRISMA guidelines and was registered in PROSPERO (CRD42024496489). A comprehensive literature search was performed in Embase, MEDLINE, Cochrane Library, CINAHL, clinicaltrial.gov, and grey literature sources up to February 28, 2025. Original studies involving pediatric patients (0–18 years) with STEC-HUS and neurological complications, treated with eculizumab, were eligible. Two independent reviewers screened studies and extracted data. Seven studies were included, totaling 529 patients, of whom 135 (25.5%) developed neurological complications. Among these, 44 patients (32.5%) had received eculizumab. Meta-analysis showed a higher likelihood of receiving eculizumab therapy in patients with neurological involvement compared to those without (OR 13.03, 95% CI 4.40–38.75). However, in patients with neurological involvement, no clinical benefit was observed compared to those treated with standard therapies (OR 0.32, 95% CI 0.09–1.22, *p* = 0.10). *Conclusion*: Our data did not demonstrate a significant improvement in neurological outcomes for STEC-HUS patients treated with eculizumab. Findings are limited by retrospective designs and potential confounding by indication; therefore, further studies are needed.

**Table:** 

**What is Known**:
• *Neurological involvement is a major contributor to morbidity in HUS, particularly in STEC-associated forms*.
• *Eculizumab is sometimes used off-label in severe cases, although its effectiveness in this setting remains uncertain*.
**What is New**:
• *This is the first systematic review and meta-analysis specifically addressing neurological prognosis in STEC-HUS*.
• *Current evidence does not demonstrate a clear neurological benefit of eculizumab over standard therapy, highlighting the need for further studies*.

**Supplementary Information:**

The online version contains supplementary material available at 10.1007/s00431-025-06160-2.

## Introduction

Hemolytic uremic syndrome (HUS) is a life-threatening thrombotic microangiopathy (TMA) characterized by the triad of non-autoimmune hemolytic anemia, thrombocytopenia, and acute kidney injury (AKI). Among its various forms, Shiga toxin-producing *Escherichia coli* (STEC)-associated HUS, or STEC-HUS, is the most common among children and a leading cause of pediatric intrinsic AKI [1]. This condition is linked to morbidity and mortality, especially when accompanied by extra-renal complications [2].

The pathogenesis of STEC-HUS begins with the production of Shiga toxin (Stx) by enterohemorrhagic *E. coli*. After entering the bloodstream, the toxin binds to globotriaosylceramide (Gb3) receptors on endothelial cells, primarily targeting the kidneys but also affecting other organs such as the brain. This interaction inhibits protein synthesis, induces endothelial apoptosis, and promotes a pro-thrombotic state, culminating in TMA [3].

While kidney involvement is the hallmark of HUS, neurological complications occur in 17–34% of affected children. These manifestations, ranging from mild cognitive changes to severe outcomes like seizures, altered mental status, or coma, result from thrombotic events in the cerebral microvasculature, leading to ischemic injury [2]. Neurological involvement is a critical determinant of poor prognosis, with survivors often facing long-term disabilities [4].

Current treatment strategies of STEC-HUS are primarily supportive. These include fluid resuscitation, correction of electrolyte imbalances, blood pressure management, kidney replacement therapy (KRT), and blood transfusions. The role of antibiotics remains controversial due to the potential for exacerbating toxin release [5].

Emerging evidence highlights the role of complement activation in the pathogenesis of STEC-HUS. Stx can activate the alternative complement pathway, exacerbating endothelial damage and intensifying TMA. This is supported by observations of elevated complement breakdown products (C3b, iC3b, C3c), reduced C3 and C4 levels, and glomerular deposition of C3 and C5b-9 during the active phase of the disease [5]. These insights have sparked interest in complement-targeted therapies, particularly eculizumab, a monoclonal antibody that inhibits complement component C5, preventing the formation of the membrane attack complex (MAC) and subsequent complement-mediated damage.

Although eculizumab has demonstrated efficacy in atypical HUS, its application in STEC-HUS remains an area of active investigation. Small observational studies have shown promising results, particularly in reducing kidney complications [6]. However, data on its effectiveness in mitigating neurological outcomes remain inconclusive. Most studies have primarily focused on kidney endpoints, leaving a gap in understanding its potential role to address neurological complications [4]. A recent randomized controlled trial (RCT) explored the use of eculizumab in pediatric STEC-HUS, finding no significant reduction in the need of KRT during the acute phase, but suggesting potential benefits for long-term kidney recovery [7]. However, neurological outcomes were not comprehensively assessed, underscoring the need for further research.

Given the high morbidity and mortality associated with severe neurological complications in STEC-HUS, this study aims to evaluate the available evidence on the impact of eculizumab treatment in improving neurological outcomes in affected children, specifically regarding symptom resolution and long-term sequelae.

## Materials and methods

### Study design

This systematic review and meta-analysis were conducted in accordance with the Preferred Reporting Items for Systematic Reviews and Meta-Analyses (PRISMA) guidelines [8]. The review protocol was registered in the PROSPERO International Prospective Register of Systematic Reviews (Registration Number CRD 42024496489). The only deviation from the registered protocol was the extension of the literature search period, which was originally planned through October 2023.

### Search strategy

We performed a comprehensive search of Embase, MEDLINE, and the Cochrane Library databases for relevant studies, up to February 28, 2025. In addition, we also performed an expanded search of specialized databases (CINAHL), clinical trial registries (clinicaltrial.gov), and grey literature (Google Scholar, OpenGrey). The search terms used were “typical hemolytic-uremic syndrome” OR “STEC-HUS” AND “eculizumab” AND “child” OR “pediatric.” Detailed search strategies were provided in Supplementary Material S1. No restrictions were applied in terms of year of publication or language. However, non-English studies were later excluded. In addition, the SCOPUS database was searched to ensure no relevant studies were missed. The reference lists of identified systematic reviews were also screened for relevant studies. Systematic reviews, narrative reviews, case reports, case series, and conference abstracts were excluded a priori. Articles considered for inclusion were original studies.

### Eligibility criteria

Eligible studies included pediatric patients (0–18 years) diagnosed with STEC-HUS, with documented neurological complications in some participants, and eculizumab administered as part of the treatment regimen. STEC-HUS was defined as presence of AKI (according to the most recent KDIGO guidelines [9]), non-autoimmune microangiopathic hemolytic anemia (hemoglobin < 10 g/dL with schistocytes on peripheral blood smear), and thrombocytopenia (platelet count < 150.000/mm^3^), with suspected or proven STEC infection. Neurological involvement was defined by the presence of clinical symptoms and/or radiological findings on brain MRI and/or electroencephalographic abnormalities. Neurological outcomes were evaluated descriptively and, when available, using the Pediatric Cerebral Performance Category (PCPC) score [10].

### Study selection

Following PRISMA guidelines, two independent reviewers (RS and AA) screened the titles and abstracts, followed by full-text assessment, to determine eligibility. Any disagreements were resolved through discussion with a third reviewer (MM). The reviewers were not blinded to the study authors, journal name, or institutions. The same inclusion and exclusion criteria were applied consistently to both the systematic review and the meta-analysis.

### Data extraction

Data extraction was performed by two authors (MM and RS) independently using a standardized form designed for this review as outlined in the protocol. Data extracted included study characteristics, participant demographics, specifics of neurological involvement, details of the intervention (eculizumab), and neurological outcomes. Additionally, data on hematological and nephrological outcomes were collected when available. Discrepancies in data interpretation were resolved through collegial discussion with a third reviewer (AA).

### Risk of bias assessment

The risk of bias for observational studies was assessed using the National Institutes of Health (NIH) risk of bias tool for observational studies. Based on study type, different versions of the NIH tool were applied. The studies were rated as “good,” “fair,” or “poor” based on a scoring system: tools with 14 questions were classified as “poor” (score 0–5), “fair” (score 6–10), and “good” (score 11–14), while tools with 12 questions followed similar criteria (poor, 0–4; fair, 5–8; good, 9–12).

### Statistical analysis

The meta-analysis was performed using RevMan 5.4 software. Risk ratios (RRs) with 95% confidence intervals (CIs) were calculated for categorical data. Heterogeneity across studies was assessed using the *I*^2^ statistic and Chi-squared test. An *I*^2^ value > 50% indicated substantial heterogeneity, prompting the use of a random-effects model for data pooling. If heterogeneity was low (*I*^2^ ≤ 50%), a fixed-effects model was applied. Publication bias was assessed using funnel plots, with symmetrical plots indicating a low likelihood of bias. In cases where publication bias was suspected, possible explanations, such as small-study effects, were discussed.

## Results

### Study selection

**Fig. 1:**
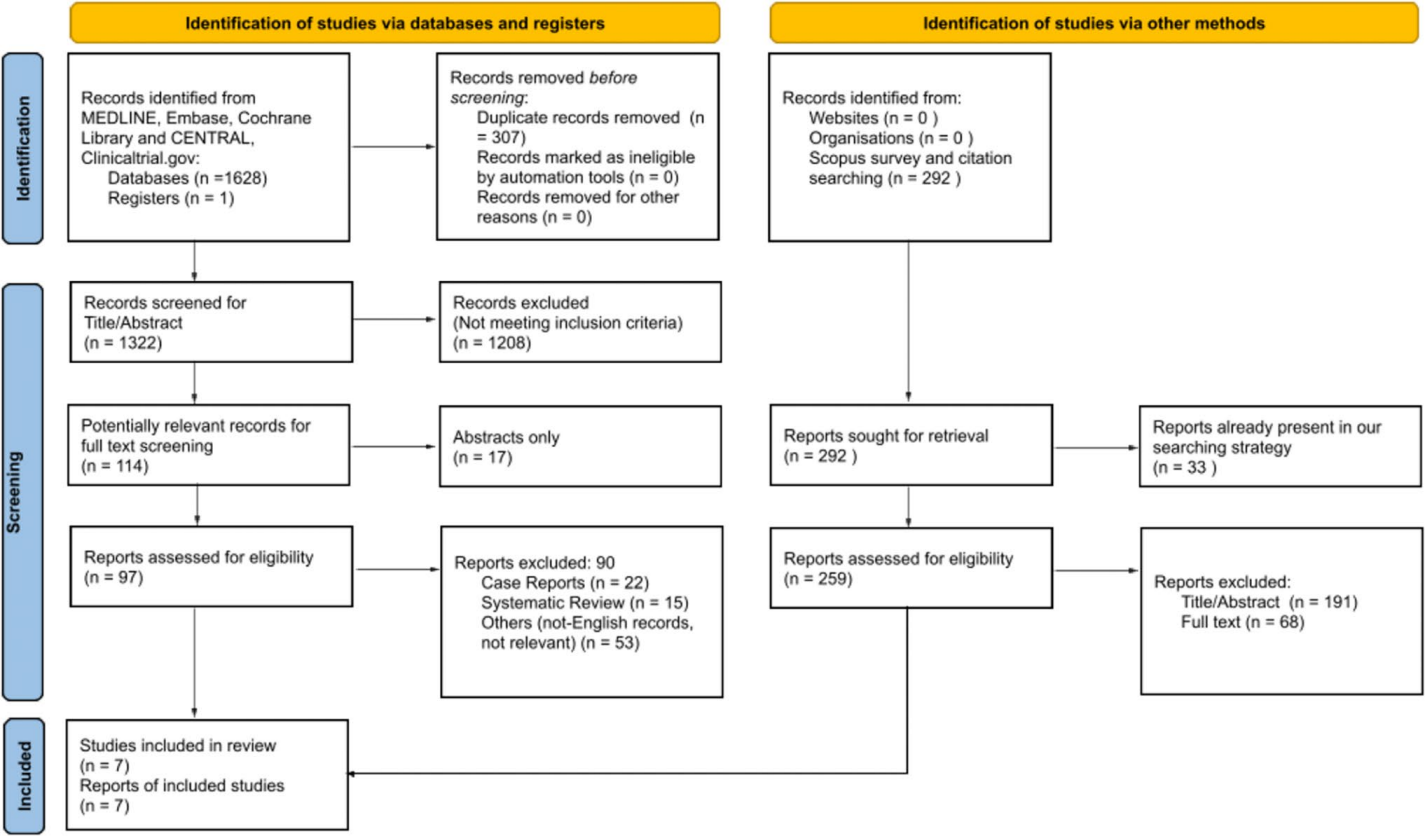
PRISMA 2020 flowchart for selection of studies

The initial search process identified a total of 1629 articles. After removing 307 duplicates using a reference management software, 1208 articles were excluded based on title and abstract screening due to not meeting inclusion criteria. Full-text reviews were conducted for 97 studies, excluding 17 that were available as abstracts only. Twenty-two case reports, 15 systematic reviews, and 53 were original articles that were excluded either because they were non-English or did not pertain to the research question. No additional studies were identified through the SCOPUS database search. Ultimately, seven studies were considered eligible for data extraction [11–17], all conducted in European settings between 2012 and 2020. Each study employed a retrospective cohort design; two were multicentric, while the remaining five were single-center studies. The study selection process is detailed in Fig. 1, and Table 1 summarizes the characteristics of the included studies.

**Table 1:** Selected studies meeting inclusion criteria

Authors	Year of publication	Journal	Country	Design of the study	Single or multicenter
Ağbaş et al. [11]	2018	Pediatric Nephrology	Turkey	Retrospective cohort study	Multicentric
Costigan et al. [12]	2021	European Journal of Pediatrics	Ireland	Retrospective cohort study	Monocentric
Giordano et al. [13]	2018	Pediatric Nephrology	Italy	Retrospective cohort study	Monocentric
Konopasek et al. [14]	2020	Klinische Padiatrie	Czech Republic	Retrospective cohort study	Monocentric
Loos et al. [15]	2012	Clinical Infectious Diseases	Germany	Retrospective cohort study	Multicentric
Monet-Didailler et al. [16]	2019	Nephrology Dialysis Transplantation	France	Retrospective cohort study	Monocentric
Ylinen et al. [17]	2020	Pediatric Nephrology	Finland	Retrospective cohort study	Multicentric

### Population description

The characteristics of the study population are summarized in Table 2. The serotypes of *E. coli* most frequently reported were EHEC O157 and O26, though incomplete data prevented a clear identification of the predominant serotype across studies. Only 42% (3/7) of the studies included patients with a clinical and microbiological diagnosis of STEC-HUS; the remaining studies classified STEC-HUS cases based solely on clinical and laboratory criteria. Most patients were younger than 5 years, except in the study by Loos et al. [15], which included a cohort with a median age of 11.5 years. A total of 529 patients were included, of whom 135 (25.5%) developed neurological complications ranging from mild irritability to seizures, stupor, and coma. Eculizumab was administered to 59 patients (11.1%), while 470 (88.9%) did not receive the drug. Among those treated with eculizumab, 44 patients exhibited some degree of neurological involvement.

**Table 2:** Characteristics of the selected studies and of the population. NI +, patients with neurological involvement; NI −, patients without neurological involvement; ECU +, patients receiving eculizumab; ECU −, patients not receiving eculizumab; NR, not reported

Authors	*E. coli* serotype (%)	Age (median [range])	Sex (%)	Definition of neurological involvement	Patients included (*N*)	NI + (*N*)	ECU + (*N*)	ECU − (*N*)	NI + ECU + (*N*)	NI + ECU − (*N*)	NI − ECU + (*N*)	NI + ECU + with neurological improvement (*N*)	NI + ECU − with neurological improvement (*N*)
Agbas et al. [11]	O104 (70%) O157 (30%)	3.25 years [0.9–14.2]	F (65) M (35)	Not clearly defined; only general mention of “'neurological manifestations”	32	8	9	23	5	3	4	4	3
Costigan et al. [12]	O157 (50%) O26 (30%) Other (20%)	3.2 years [1.6–6.3]	NR	Encephalopathy, focal neurological deficit and/or seizure activity	202	22	8	194	8	14	0	6	11
Giordano et al. [13]	O26 (53%) O145 (18%) O111 (15%) O153 or O107 (7%)	2.6 years [0.6–12.9]	F (57) M (43)	≥ 1 clinical sign, EEG or imaging	54	12	5	49	5	7	0	4	7
Konopasek et al. [14]	O26 (23%) Other (67%)	4.5 years [0.8–14]	F (70) M (30)	Severe complications: unconsciousness, seizures or cerebral edema	10	10	4	6	4	6	0	3	3
Loos et al. [15]	O104 (% NR)	11.5 years [0.6–17.5]	F (54) M (46)	No specific definition: mainly seizures and altered mental status	90	23	13	77	10	13	3	NR	NR
Monet-Didailler et al. [16]	NR	3.4 years [1.3–9.2]	F (61) M (39)	No specific definition	54	19	18	36	10	9	6	4	8
Ylinen et al. [17]	NR	NR	F (63) M (37)	“Major neurological symptoms,” no further detail	87	41	2	85	2	39	0	1	38
**Total, *N* (%)**					529 (100)	135 (25.5)	59 (11.1)	470 (88.9)	44	91	13	22	70

### Risk of receiving eculizumab according to the presence of neurological involvement

**Fig. 2:**
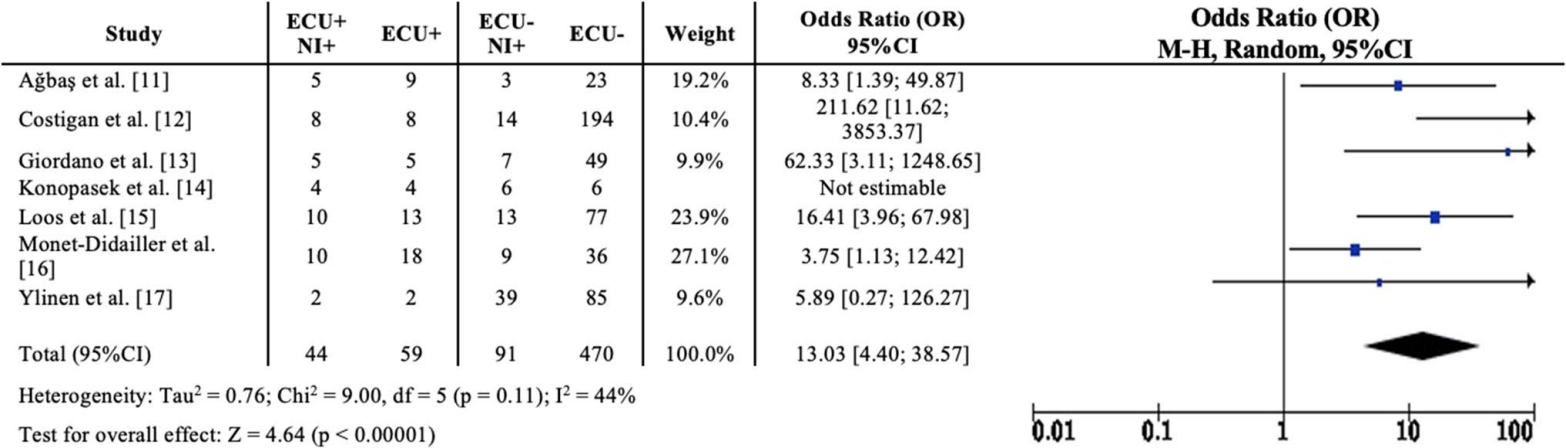
Risk of receiving eculizumab according to the presence of neurological involvement*.* NI +, patients with neurological involvement; ECU +, patients receiving eculizumab; ECU −, patients not receiving eculizumab

The analysis demonstrated a higher likelihood of receiving eculizumab among patients with neurological involvement with an odds ratio (OR) of 13.03 (95% CI 4.40–38.57) compared to those without neurological symptoms, with moderate heterogeneity (*I*^2^ = 44%) and statistically significant results (*z* = 4.64, *p* < 0.00001). In the eculizumab-treated group, 44 out of 59 patients (74.5%) exhibited neurological involvement, whereas in the non-treated group, 91 out of 470 patients (19.3%) showed similar symptoms. Figure 2 presents the comparison of neurological involvement between the eculizumab-treated and untreated groups. The study by Konopasek et al. [14] was excluded due to the absence of data on patients without neurological involvement, thereby lacking a comparator group for analysis.

### Neurological improvement following eculizumab administration

**Fig. 3:**
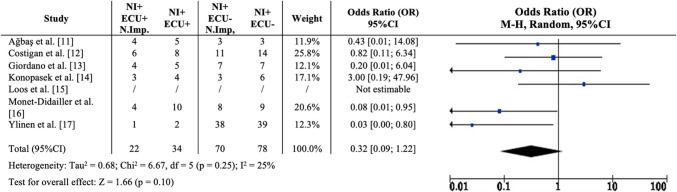
Likelihood of achieving an improved neurological outcome following eculizumab treatment. NI +, patients with neurological involvement; ECU +, patients receiving eculizumab; ECU −, patients not receiving eculizumab. N.Imp., neurological improvement

Among patients with neurological involvement, 64% (22/34) in the eculizumab-treated group demonstrated neurological improvement, compared to 89% (70/78) in the non-eculizumab group. The overall OR for improvement was 0.32 (95% CI 0.09–1.22), with low heterogeneity (*I*^2^ = 25%). However, the difference between the two groups was not statistically significant (*z* = 1.66, *p* = 0.10). The study by Loos et al. [15] was excluded from this analysis due to the unavailability of data on neurological outcomes, which precluded the identification of a control group. The likelihood of achieving an improved neurological outcome following eculizumab treatment is illustrated in Fig. 3.

### Quality assessment

**Fig. 4:**
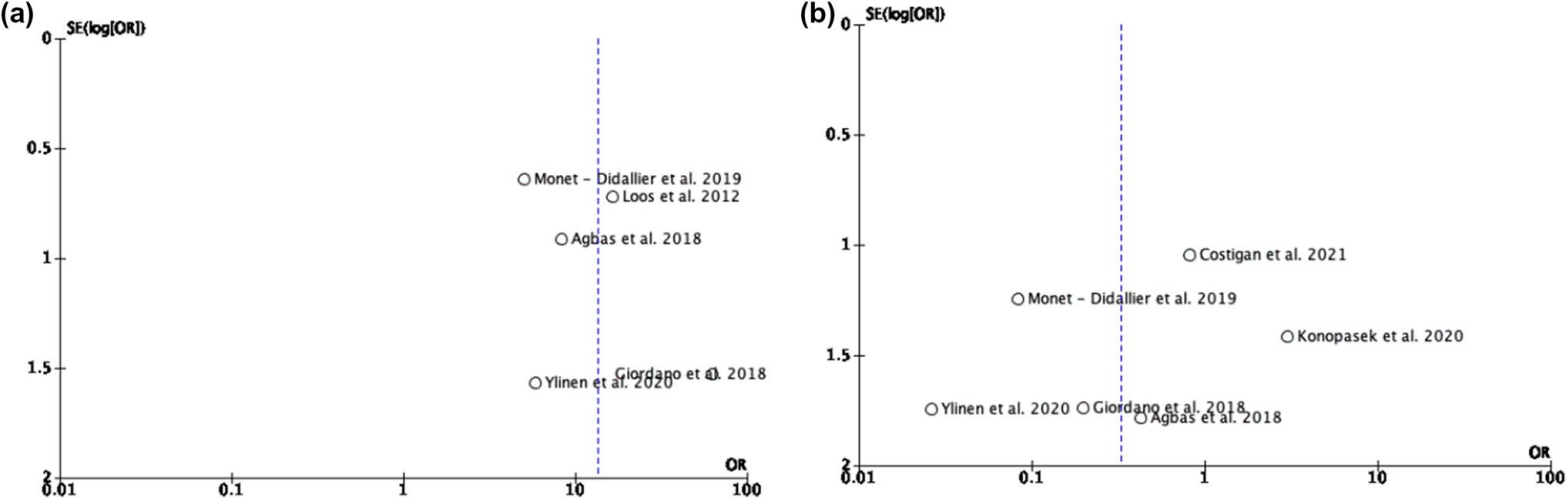
Funnel plot evaluating publication bias for the outcome “association between neurological involvement and eculizumab administration” (**a**) and “neurological involvement following eculizumab administration” (**b**)

The risk of bias for the included observational studies was assessed using the NIH risk of bias tool (see Supplementary Material S2 and Table 3). In addition, to explore potential publication bias, we constructed two funnel plots, each corresponding to one of the analyzed outcomes (Fig. 4). For the outcome “association between neurological involvement and eculizumab administration” (Fig. 4a), the funnel plot exhibits significant asymmetry, with a right-skewed distribution of studies, suggesting a potential publication bias favoring studies reporting positive results. This pattern indicates that studies with significant findings may have been preferentially published. In contrast, for the outcome “neurological involvement following eculizumab administration” (Fig. 4b), the funnel plot shows a more balanced distribution of studies around the reference line, suggesting a lower risk of publication bias. However, some residual right-skewed asymmetry is still present, which may indicate a tendency for studies with non-significant results to remain unpublished.

**Table 3:**
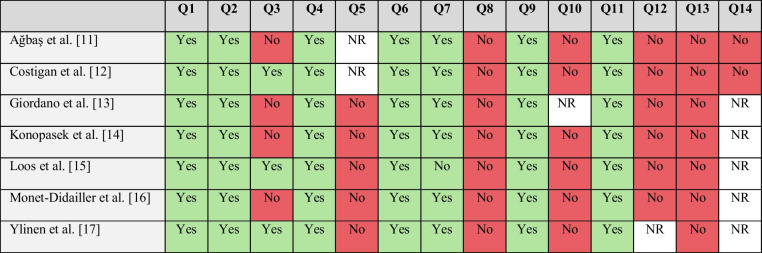
Application of the NIH risk of bias tool to assess study quality. NR, not reported

**Fig. 5:**
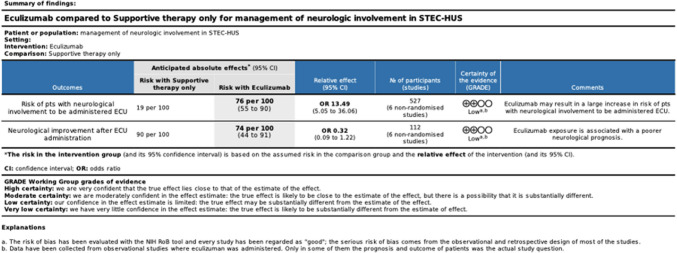
Summary of findings with GRADE criteria

Using the GRADE (Grading of Recommendation, Assessment, Development and Evaluation) approach, we rated the overall certainty of the evidence for each outcome analyzed in the meta-analysis as low. This rating reflects observational nature of all included studies, along with concerns regarding risk of bias, indirectness, and imprecision. Inconsistency was not considered a serious issue, as the meta-analysis results were generally consistent and showed only moderate heterogeneity. However, indirectness was a notable limitation, since most studies did not directly evaluate the effect of eculizumab on disease progression or neurological outcomes. Imprecision was rated as not serious, with clearly defined outcome measures across studies. Nevertheless, the certainty of evidence was further limited by potential confounding factors, particularly due to the rapid and acute progression of the disease, which might have affected treatment timing and efficacy. The summary of GRADE ratings is presented in Fig. 5.

## Discussion

This systematic review and meta-analysis aimed to evaluate the role of eculizumab in improving neurological outcomes in pediatric patients with severe STEC-HUS. Although eculizumab has demonstrated efficacy in atypical HUS (aHUS), its potential benefit in STEC-HUS remains uncertain and appears to depend on the specific clinical context, with current evidence being limited and inconsistent [6].

Our meta-analysis revealed a significantly higher likelihood of eculizumab administration in children with neurological involvement compared to those without such complications. This trend aligns with clinical practices where eculizumab is often prioritized for severe cases, including those with neurological manifestations, due to its role in mitigating complement-mediated endothelial damage [18]. This association, however, was accompanied by considerable variability, as indicated by a wide confidence interval, suggesting uncertainty in the strength of this relationship.

While reports suggest clinical improvements in STEC-HUS cases with neurological complications, the available evidence is derived predominantly from retrospective studies and case reports, which raises concerns about publication bias [6, 18]. For example, Mahat et al. noted that 15 out of 21 patients with neurological involvement showed rapid improvement following eculizumab treatment [18]. Conversely, larger studies in adult cohorts, such as the open-label trial by Kielstein et al., reported no added benefit of eculizumab over standard supportive care or plasma exchange, even after adjusting for confounders [19].

Our findings corroborate the challenges highlighted in prior research. Specifically, patients with neurological involvement treated with eculizumab showed less improvement compared to those receiving standard therapies, although the difference did not reach statistical significance. This disparity may reflect the greater severity of clinical presentations in patients selected for eculizumab, as well as inconsistencies in reporting the timing of its administration, both of which could significantly impact its efficacy.

We acknowledge the recent publication of the ECUSTEC randomized controlled trial, which assessed the efficacy of eculizumab in pediatric STEC-HUS [20]. Although the study could not be included in our meta-analysis—since its outcomes were reported as part of a composite severity score, preventing the extraction of data specific to neurological involvement—it represents an important addition to the existing literature. Notably, despite early termination and limited statistical power, the trial found no significant difference between eculizumab and placebo groups in terms of persistent neurological deficits at day 60, a finding consistent with our results and further underscoring the current lack of clear evidence supporting a neurological benefit of eculizumab in this context.

The findings highlight important considerations for managing pediatric STEC-HUS with neurological complications. While eculizumab may offer benefits for specific subgroups, current evidence does not support its routine use to improve neurological outcomes. Supportive care, including fluid resuscitation, blood pressure management, and kidney replacement therapy, continued to be the cornerstone of treatment [1]. The high cost of eculizumab further limits its feasibility, particularly in resource-constrained settings, where cost-effectiveness is a paramount concern. In this context, our finding may provide reassurance to clinicians working in low-resource environments, reinforcing that the absence of eculizumab does not appear to compromise neurological outcomes when high-quality supportive care is provided. The recent availability of biosimilar forms could, however, offer a lower-cost therapeutic option, potentially expanding access. In weighing the risk and benefits of eculizumab, it is important to consider its safety profile. By blocking complement component 5, eculizumab increases susceptibility to infections, particularly those caused by encapsulated organisms. To mitigate these risks, appropriate vaccination and short-term antibiotic prophylaxis are recommended prior to treatment [3, 5]. Despite these challenges, the prognosis in patients with neurological involvement often dictates treatment priorities. Considering the limited alternative therapies, clinicians must carefully weigh the benefits and risks of eculizumab on a case-by-case basis.

Our findings should be considered in the context of certain limitations. Despite implementing a comprehensive search strategy and manually reviewing references to include all relevant data, it is not possible to completely rule out the existence of other studies that were not included in this review. Another important consideration is the overall quality of the included studies. Most of the studies analyzed were retrospective, introducing potential biases such as selection bias, variations in patient management, and inconsistencies in outcome reporting. The lack of RCTs limits control over confounding factors, particularly regarding disease severity at onset and the timing of eculizumab administration. Importantly, the absence of a significant benefit in neurological outcomes should not be interpreted as evidence of ineffectiveness of eculizumab; rather, it may reflect confounding by indication, whereby eculizumab was more frequently administered to patients with more severe disease, potentially obscuring any treatment effect. Additionally, small sample sizes and short follow-up durations restrict the ability to assess long-term neurological outcomes. Variability in diagnostic criteria and neurological assessments across studies further complicates result comparability.

The NIH risk of bias assessment indicated varying levels of methodological rigor, with concerns regarding selection bias and inconsistencies in outcome reporting. The heterogeneity in diagnostic criteria and neurological outcome assessment across studies may have influenced the consistency and comparability of results. Additionally, the observed publication bias suggests that studies with negative or inconclusive results may be underrepresented in the literature, potentially affecting the overall interpretation of the findings. Another limitation was the lack of some data in certain studies. To address this, we prioritized transparency in data reporting, relying on the information available within the included studies.

This variability in neurological outcomes may also reflect limitations in the tools currently used to assess brain injury in this population. Conventional clinical and radiological evaluations may underestimate the extent of neurological involvement, particularly in the acute setting. Recent data suggest that early MRI combined with quantitative apparent diffusion coefficient (ADC) measurements may improve the detection of cerebral involvement in STEC-HUS. Bültmann et al. demonstrated that ADC alterations, indicative of microstructural brain changes, could be identified even in regions appearing normal on standard MRI sequences. Moreover, more extensive ADC abnormalities were observed in patients with poor neurological outcomes. These findings suggest that early quantitative MRI could help identify children at higher risk of persistent neurological sequelae and should be considered in the design of future interventional studies aiming to assess therapies such as eculizumab [21].

The limitations emphasize the need for high-quality RCTs focusing on eculizumab’s effects on neurological outcomes in pediatric STEC-HUS. Future research should aim to establish clear indications, optimal timing, and potential combination therapies. Long-term follow-up is also necessary to assess chronic neurological sequelae and the persistence of observed benefits. Understanding the pathophysiology of STEC-HUS further could lead to more targeted therapeutic interventions and improved patient selection criteria. Future research should focus on identifying clinical and laboratory predictors of severe neurological involvement, allowing for better selection of patients who would benefit most from specific treatment.

## Conclusions

This systematic review and meta-analysis assessed the role of eculizumab in managing severe pediatric STEC-HUS, particularly its impact on neurological outcomes. Our findings suggest that while eculizumab is more commonly used in patients with neurological complications, its efficacy in improving neurological outcomes remains uncertain, with no significant advantage over standard supportive care observed.

The evidence is limited by the retrospective design of included studies, small sample sizes, and a lack of RCTs focusing on neurological outcomes. These limitations highlight the need for further well-designed research to determine the optimal use of eculizumab, including timing and patient selection, and to better understand its long-term effects.

## Supplementary Information

Below is the link to the electronic supplementary material.

Supplementary file1 (DOCX 17 KB)

## Data Availability

No datasets were generated or analysed during the current study.

## Abbreviations

aHUS: Atypical hemolytic uremic syndrome
AKI: Acute kidney injury
CI: Confidence interval
Gb3: Globotriaosylceramide
GRADE: Grading of Recommendation, Assessment, Development and Evaluation
HUS: Hemolytic uremic syndrome
KRT: Kidney replacement therapy
MAC: Membrane attack complex
NIH: National Institutes of Health
OR: Odds ratio
PCPC: Pediatric Cerebral Performance Category
PRISMA: Preferred Reporting Items for Systematic Reviews and Meta-Analyses
RCT: Randomized controlled trial
RR: Risk ratio
STEC: Shiga toxin-producing *Escherichia coli*
Stx: Shiga toxin
TMA: Thrombotic microangiopathy
