# Supplementary material for: Eculizumab in severe pediatric STEC-HUS and its impact on neurological prognosis—a systematic review and meta-analysis
Source: Eur J Pediatr. 2025 May 8;184(6):331. doi: 10.1007/s00431-025-06160-2 (PMC12062034; doi:10.1007/s00431-025-06160-2)
Supplement: Supplementary file 1 — Supplementary file1 (DOCX 17 KB) [file 431_2025_6160_MOESM1_ESM.docx]

**Supplementary Material**

**Supplementary Material S1.** Search strategy.

*Medline*

((child[MeSH Terms]) OR (child, preschool[MeSH Terms]) OR (adolescent[MeSH Terms]) OR (infant[MeSH Terms]) OR (Infant, Newborn [MeSH Terms]) OR (Infants, Newborn[Title/Abstract]) OR (Newborn Infant[Title/Abstract]) OR (Newborn Infants[Title/Abstract]) OR (Newborn*[Title/Abstract]) OR (Neonat*[Title/Abstract]) OR (Preschool Child[Title/Abstract]) OR (Children, Preschool[Title/Abstract]) OR (Child*[Title/Abstract]) OR (Preschool Children[Title/Abstract]) OR (infant*[Title/Abstract]) OR (Adolescent*[Title/Abstract]) OR (Teen*[Title/Abstract]) OR (Youth*[Title/Abstract])) AND ((Hemolytic-Uremic Syndrome[MeSH Terms]) OR (Gasser Syndrome[Title/Abstract]) OR (Gasser's Syndrome[Title/Abstract]) OR (Gassers Syndrome[Title/Abstract]) OR (Syndrome, Hemolytic-Uremic[Title/Abstract]) OR (“Typical Hemolytic Uremic Syndrome”[Title/Abstract]) OR (“STEC-HUS”[Title/Abstract]) OR (“Typical HUS”[Title/Abstract] OR (“STEC Hemolytic Uremic Syndrome”[Title/Abstract]) AND ((shiga toxins[MeSH Terms]) OR (Escherichia coli[MeSH Terms])) OR (verotoxin[MeSH Terms)) AND (eculizumab)

*Embase*

((exp child/) OR (exp "child, preschool"/) OR (exp adolescent/) OR (exp infant/) OR (exp "Infant, Newborn"/) OR ("Infants, Newborn".tw.) OR ("Newborn Infant".tw.) OR ("Newborn Infants".tw.) OR (Newborn*.tw.) OR (Neonat*.tw.) OR ("Preschool Child".tw.) OR ("Children, Preschool".tw.) OR (Child*.tw.) OR ("Preschool Children".tw.) OR (infant*.tw.) OR (Adolescent*.tw.) OR (Teen*.tw.) OR (Youth*.tw.)) AND (exp "Hemolytic-Uremic Syndrome"/) OR ("Gasser Syndrome".tw.) OR ("Gasser's Syndrome".tw.) OR ("Gassers Syndrome".tw.) OR ("Syndrome, Hemolytic-Uremic".tw.) OR ("Typical Hemolytic Uremic Syndrome".tw.) OR (STEC-HUS.tw.) OR ("Typical HUS".tw. OR ("STEC Hemolytic Uremic Syndrome".tw.) AND ((exp "shiga toxins"/) OR (exp "Escherichia coli"/)) OR ("verotoxin[MeSH Terms")) AND (eculizumab)

*Cochrane*

(([mh child]) OR ([mh "child, preschool"]) OR ([mh adolescent]) OR ([mh infant]) OR ([mh "Infant, Newborn"]) OR ("Infants, Newborn":ti,ab) OR ("Newborn Infant":ti,ab) OR ("Newborn Infants":ti,ab) OR (Newborn*:ti,ab) OR (Neonat*:ti,ab) OR ("Preschool Child":ti,ab) OR ("Children, Preschool":ti,ab) OR (Child*:ti,ab) OR ("Preschool Children":ti,ab) OR (infant*:ti,ab) OR (Adolescent*:ti,ab) OR (Teen*:ti,ab) OR (Youth*:ti,ab)) AND ([mh "Hemolytic-Uremic Syndrome"]) OR ("Gasser Syndrome":ti,ab) OR ("Gasser's Syndrome":ti,ab) OR ("Gassers Syndrome":ti,ab) OR ("Syndrome, Hemolytic-Uremic":ti,ab) OR ("Typical Hemolytic Uremic Syndrome":ti,ab) OR (STEC-HUS:ti,ab) OR ("Typical HUS":ti,ab OR ("STEC Hemolytic Uremic Syndrome":ti,ab) AND (([mh "shiga toxins"]) OR ([mh "Escherichia coli"])) OR ("verotoxin[MeSH Terms")) AND (eculizumab)

**Supplementary Material S2.** *Quality Assessment Tool for Observational Cohort and Cross-Sectional Studies*

*(https://www.nhlbi.nih.gov/health-topics/study-quality-assessment-tools, last accessed October 15, 2022)*

| **Criteria** | **Yes** | **No** | **Other**  **(CD, NR, NA)*** |
| --- | --- | --- | --- |
| 1. Was the research question or objective in this paper clearly stated? |  |  |  |
| 2. Was the study population clearly specified and defined? |  |  |  |
| 3. Did the literature search strategy use a comprehensive, systematic approach? |  |  |  |
| 4. Were all the subjects selected or recruited from the same or similar populations (including the same time period)? Were inclusion and exclusion criteria for being in the study prespecified and applied uniformly to all participants? |  |  |  |
| 5. Was a sample size justification, power description, or variance and effect estimates provided? |  |  |  |
| 6. For the analyses in this paper, were the exposure(s) of interest measured prior to the outcome(s) being measured? |  |  |  |
| 7. Was the timeframe sufficient so that one could reasonably expect to see an association between exposure and outcome if it existed? |  |  |  |
| 8. For exposures that can vary in amount of level, did the study examine different levels of the exposure as related to the outcome (e.g., categories of exposure, or exposure measured as continuous variable)? |  |  |  |
| 9. Were the exposure measures (independent variables) clearly defined, valid, reliable, and implemented consistently across all study participants? |  |  |  |
| 10. Was the exposure(s) assessed more than once over time? |  |  |  |
| 11. Were the outcome measures (dependent variables) clearly defined, valid, reliable, and implemented consistently across all study participants? |  |  |  |
| 12. Were the outcome assessors blinded to the exposure status of participants? |  |  |  |
| 13. Was loss to follow-up after baseline 20% or less? |  |  |  |
| 14. Were key potential confounding variables measured and adjusted statistically for their impact on the relationship between exposure(s) and outcome(s)? |  |  |  |

*CD, cannot determine; NA, not applicable; NR, not reported
